# Intravesicle Isothermal DNA Replication

**DOI:** 10.1186/1756-0500-4-128

**Published:** 2011-04-15

**Authors:** Domenica Torino, Cristina Del Bianco, Lindsey A Ross, Jennifer L Ong, Sheref S Mansy

**Affiliations:** 1 Centre for Integrative Biology, University of Trento, Via delle Regole, 101 Mattarello, Italy; 2 New England BioLabs, 240 County Road, Ipswich, Massachusetts 01938-2723, USA; 3 School of Pharmacy, University of Colorado Denver, Aurora, Colorado 80045, USA

## Abstract

**Background:**

Bacterial and viral DNA replication was previously reconstituted *in vitro *from component parts [1-4]. Significant advances in building minimal cell-like structures also have been made recently [5-7]. Combining the two approaches would further attempts to build a minimal cell-like structure capable of undergoing evolution by combining membrane encapsulation and genome replication. Towards this end, we attempted to use purified genomic replication protein components from thermophilic bacterial sources to copy strands of DNA isothermally within lipid vesicles.

**Findings:**

Bacterial replication components (such as helicases and DNA polymerases) are compatible with methods for the generation of lipid vesicles. Encapsulation inside phospholipid vesicles does not inhibit the activity of bacterial DNA genome replication machinery. Further the described system is efficient at isothermally amplifying short segments of DNA within phospholipid vesicles.

**Conclusions:**

Herein we show that bacterial isothermal DNA replication machinery is functional inside of phospholipid vesicles, suggesting that replicating cellular mimics can be built from purified bacterial components.

## Findings

Much interest has centred on building encapsulated replicating genetic systems capable of Darwinian evolution [5,6]. Typically, the exploited methods are based on PCR, including DNA replication within water-in-oil (w/o) emulsions and phospholipid vesicles [8-10]. Thermocycling methods are undesirable for the construction of cell-like structures since they generate non-autonomous systems, i.e. intervention is required for the cycling of temperature. An alternative is to exploit previously constructed *in vitro *isothermal DNA amplification methods [1,2,11]. Since extant life uses compartments defined by lipid bilayers, we sought to reconstitute an isothermal replication system inside of phospholipid vesicles. We did not exploit fatty acid vesicles, because fatty acid vesicles are less stable [12] and previous work has shown an incompatibility between fatty acids and some DNA polymerases [13].

To build an isothermal DNA replication system inside of vesicles, several features are desirable. The system must survive mechanisms of vesicle generation, be functional within the microenvironment of the vesicle compartment, and be controllable so that reactions only occur after encapsulation. We find that the previously described tHDA (thermophilic helicase-dependent amplification) system [1,2] possesses all of these features, suggesting that the reconstitution of bacterial machinery may provide for a facile route towards the building of replicating cell-like structures. The tHDA mix of thermostable proteins includes a UvrD helicase, a single-strand binding protein, and a DNA polymerase.

## Results and discussion

**Figure 1:**
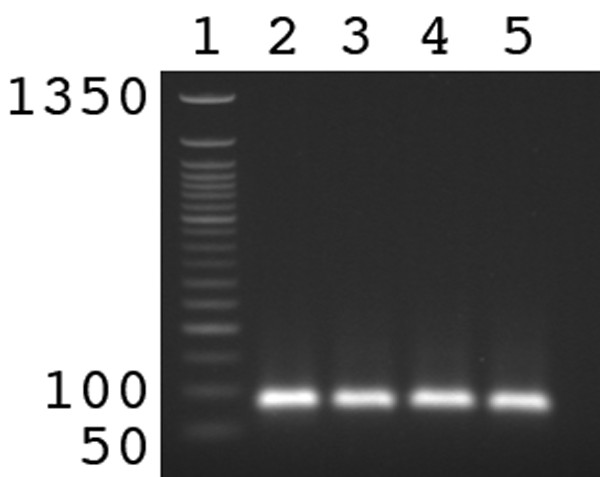
**The influence of freeze/thaw cycles on tHDA enzymatic activity**. Unencapsulated reaction mixtures were subjected to either 0 (lane 2), 5 (lane 3), 10 (lane 4), or 20 (lane 5) cycles of freeze-thawing. Lane 1 is a 50 bp DNA ladder. The 1350 bp, 100 bp, and 50 bp bands are labeled. Reaction products were visualized by ethidium bromide staining of a 1.8% agarose gel. The full length reaction product is 85 bp.

To test whether the previously described tHDA system [1,2] functions after cycles of freeze/thawing, we subjected aliquots of tHDA to up to 20 cycles of freezing on dry ice followed by thawing at 30°C. Subsequently, the solutions were incubated at 65°C to allow for the thermophilic DNA polymerase to replicate the DNA template. As seen in Figure 1, up to 20 cycles of freeze/thawing did not inhibit the reaction. This is useful because freeze/thaw cycles are a common method to increase encapsulation efficiency and to facilitate the formation of vesicles [14,15].

**Figure 2:**
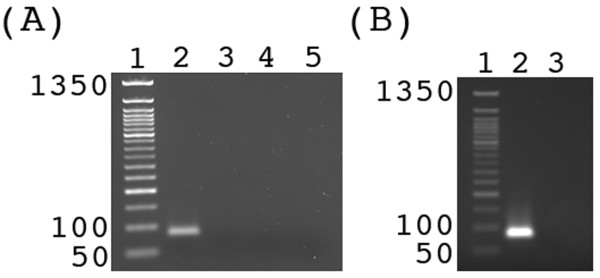
**The effect of temperature on tHDA enzymatic activity**. (A) The influence of temperature on the enzymatic activity of the isothermal tHDA system. Samples were incubated at 65°C (lane 2), 37°C (lane 3), 23°C (lane 4), and 4°C (lane 5) for 1.5 h. Lane 1 contains a 50 bp DNA ladder. The 1350 bp, 100 bp, and 50 bp bands are labeled. (B) The influence of overnight incubation temperature on the enzymatic activity of the isothermal tHDA system. Samples were incubated at 4°C (lane 2) and 23°C (lane 3) overnight prior to incubation at 65°C for 1.5 h. Lane 1 is a 50 bp DNA ladder. Reaction products were observed by ethidium bromide staining of a 1.8% agarose gel.

Next, we wanted to ensure that the reaction was controllable by temperature. Since all of the proteins of the tHDA system are from thermophilic microorganisms, we expected a greatly diminished ability to replicate DNA at low temperatures. Therefore, we tested the activity of the tHDA system at 4°C, 23°C, 37°C, and 65°C. As expected, the yield at all of the tested temperatures, except for 65°C, was below the detection limit (<5 ng) of ethidium bromide staining of an agarose gel (Figure 2A). This not only allows for the control of DNA replication by temperature, but also facilitates preparatory steps, including those of vesicle generation and the enzymatic degradation of extravesicular material.

Since vesicle production methods typically employ an overnight incubation step, we tested the ability of the tHDA system to survive overnight incubation. The reaction components were mixed on ice and then either incubated overnight at 4°C or 23°C followed by an incubation at 65°C to allow the system to replicate DNA. We could not detect amplification after an overnight incubation at 23°C. Conversely, incubation at 4°C overnight did not observably diminish DNA yields (Figure 2B).

**Figure 3:**
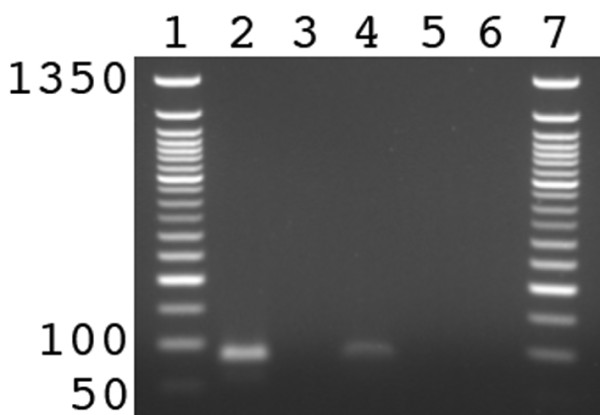
**Intravesicular isothermal replication**. Lanes 1 and 7 contain a 50 bp DNA ladder. Lanes 2 and 3 are from unencapsulated reactions and lanes 4-6 are within phospholipid vesicles. Lanes 2 and 3 are isothermal replication reactions under standard conditions except that 0.5 mM CaCl_2 _was added. CaCl_2 _is needed for proteinase K activity. Further, lane 3 contained 0.9 units of proteinase K, demonstrating that proteinase K is capable of digesting components of the tHDA system and thus inhibiting DNA amplification. A similar experiment was conducted inside of vesicles with proteinase K added to the outside of the vesicles (lane 4) and proteinase K added to both the inside and the outside of the vesicles (lane 5). Finally, to further demonstrate that the reaction was encapsulated, dNTPs were added extravesicularly resulting in an inability to replicate DNA since dNTPs are incapable of crossing POPC membranes (lane 6).

Having established that the tHDA system is controllable and survives the steps necessary for vesicle formation, we encapsulated the tHDA system in POPC (1-palmitoyl-2-oleoyl-sn-glycero-3-phosphocholine) vesicles. The protocol exploited an overnight incubation at 4°C of the tHDA components with phospholipids, 20 freeze/thaw cycles, an incubation with proteinase K that was added to the outside of the vesicles to inhibit extravesicular reactions, and finally incubation at 65°C for 1.5 h. As seen in Figure 3, the isothermal amplification of DNA occurred within the phospholipid vesicles. The presence of proteinase K outside of the vesicles did not inhibit the reaction, whereas the inclusion of the protease in both the intra- and extra-vesicle environment inhibited DNA amplification. As a further confirmation that the reaction occurred inside of the vesicles, dNTPs were added outside, but not inside, of the vesicles. Since POPC membranes are impermeable to nucleotides [16], the replication reaction was undetectable. The lower band intensity resulting from the intravesicle reaction shown in Figure 3 reflects the inefficiency of the overall reaction. For example, based on total sample volumes, the vesicle reactions were only ca. 1% as efficient as the solution reactions. However, such a comparison is misleading. The total intravesicle volume is much lower than the total solution volume. Further, a functional compartment requires the simultaneous encapsulation of several components, including a template, two primers, and three proteins. Similar difficulties arising from the Poisson distribution of reaction components within vesicles have been thoroughly described by Luisi [10] and Yomo [17,18]. Nevertheless, the efficiency of the encapsulated tHDA system is sufficient, in the sense that only a single functional compartment is required to build a self-replicating cell-like structure capable of propagation.

In summary, it is possible to reconstitute bacterial DNA replication machinery capable of copying DNA isothermally inside of phospholipid vesicles.

## Materials and methods

### Materials

The IsoAmp tHDA kit and 50 bp DNA ladder were from New England BioLabs. 1-palmitoyl-2-oleoyl-sn-glycero-3-phosphocholine (POPC) was obtained from Genzyme, and proteinase K was purchased from Fermentas Life Sciences. All other chemicals were from Sigma-Aldrich.

### Freeze/thaw stability of protein components

The reactions were prepared and performed as described in the manufacturer's instructions. The template was the control DNA provided by the kit. Reactions were performed in the absence of lipids. Each sample was subjected to a differing number of freeze-thaw cycles in which one cycle corresponded to 2 min on dry ice followed by 2 min at 30°C. 50 μL isothermal reactions were run at 65 °C for 90 min in a MJ Mini Thermal Cycler (Bio-Rad). A 10 μL aliquot of each reaction was mixed with loading buffer and separated on a 1.8% agarose gel with TBE (Tris/Borate/EDTA) as the running buffer. Ethidium bromide gels were visualized with either a BioDoc-It Imaging System (UVP) or a Molecular Imager ChemiDoc XRS System (Bio-Rad).

### Influence of Temperature on tHDA activity

To test for activity at different temperatures, samples were prepared as described in the manufacturer's instructions except that aliquots were incubated at either at 4°C, 23°C, 37°C, or 65°C for 1.5 h. Aliquots were then loaded on a 1.8% agarose TBE gel and stained with ethidium bromide. To assess the overnight temperature stability of the tHDA reaction mixture, prior to incubation at 65°C to allow for DNA amplification, the samples were either incubated at 4°C or 23°C overnight. Aliquots were then loaded on a 1.8% agarose TBE gel and stained with ethidium bromide.

### Intravesicular isothermal reactions

Vesicles were prepared by the thin lipid film hydration method. Briefly, POPC was dissolved in chloroform and evaporated in a round bottom flask with a Buchi Rotavapor R-210 and a Buchi Vacuum Pump V-700. The resulting thin lipid film was hydrated with the reaction components provided by NEB except that a buffer consisting of 10 mM KCl, 10 mM (NH_4_)_2_SO_4_, 20 mM Tris-HCl, pH 8.8 was used in place of the buffer provided by the kit. At this point the volume was 100 μL and the lipid concentration was 26 mM. The solution was then vigorously vortexed and incubated on a rotisserie at 4°C overnight. Afterwards, the dispersion was subjected to 20 freeze-thaw cycles (1 cycle = 2 min on dry ice followed by 2 min at 30°C). Subsequently, the solution was incubated with 3.6 units of proteinase K at 37°C for 30 min. Finally, the isothermal reaction was carried-out by incubating the mixture at 65°C for 90 min. Reactions were stopped by phenol/chloroform extraction followed by ethanol precipitation. The pellet was resuspended in 10 μL of deionized water, mixed with loading buffer and loaded onto a 1.8% agarose gel using TBE as the running buffer. Fluorescence microscopy of aliquots stained with rhodamine 6G revealed that the average vesicle size generated by this method was 5 μm.

## Competing interests

The authors declare that they have no competing interests.

## Authors' contributions

DT, CD, and SSM designed the experiments. DT and CD performed the experiments. LAR carried-out preliminary experiments. DT, CD, JLO, and SSM analyzed the data and wrote the paper. All authors read and approved the final manuscript.
